# Detection and characterization of copy number variation in three differentially-selected Nellore cattle populations

**DOI:** 10.3389/fgene.2024.1377130

**Published:** 2024-04-17

**Authors:** Lorena F. Benfica, Luiz F. Brito, Ricardo D. do Bem, Leticia F. de Oliveira, Henrique A. Mulim, Larissa G. Braga, Joslaine N. S. G. Cyrillo, Sarah F. M. Bonilha, Maria Eugenia Z. Mercadante

**Affiliations:** ^1^ Department of Animal Sciences, Purdue University, West Lafayette, IN, United States; ^2^ Department of Animal Science, Faculty of Agricultural and Veterinary Sciences, Sao Paulo State University, Jaboticabal, São Paulo, Brazil; ^3^ Department of Animal Biosciences, University of Guelph, Guelph, ON, Canada; ^4^ Beef Cattle Research Center, Institute of Animal Science, Sertaozinho, São Paulo, Brazil

**Keywords:** beef cattle, copy number variation, gene annotation, Nellore, residual feed intake, SNP panel

## Abstract

**Introduction:** Nellore cattle (*Bos taurus indicus*) is the main beef cattle breed raised in Brazil. This breed is well adapted to tropical conditions and, more recently, has experienced intensive genetic selection for multiple performance traits. Over the past 43 years, an experimental breeding program has been developed in the Institute of Animal Science (IZ, Sertaozinho, SP, Brazil), which resulted in three differentially-selected lines known as Nellore Control (NeC), Nellore Selection (NeS), and Nellore Traditional (NeT). The primary goal of this selection experiment was to determine the response to selection for yearling weight (YW) and residual feed intake (RFI) on Nellore cattle. The main objectives of this study were to: 1) identify copy number variation (CNVs) in Nellore cattle from three selection lines; 2) identify and characterize CNV regions (CNVR) on these three lines; and 3) perform functional enrichment analyses of the CNVR identified.

**Results:** A total of 14,914 unique CNVs and 1,884 CNVRs were identified when considering all lines as a single population. The CNVRs were non-uniformly distributed across the chromosomes of the three selection lines included in the study. The NeT line had the highest number of CNVRs (*n* = 1,493), followed by the NeS (*n* = 823) and NeC (*n* = 482) lines. The CNVRs covered 23,449,890 bp (0.94%), 40,175,556 bp (1.61%), and 63,212,273 bp (2.54%) of the genome of the NeC, NeS, and NeT lines, respectively. Two CNVRs were commonly identified between the three lines, and six, two, and four exclusive regions were identified for NeC, NeS, and NeT, respectively. All the exclusive regions overlap with important genes, such as *SMARCD3*, *SLC15A1*, and *MAPK1*. Key biological processes associated with the candidate genes were identified, including pathways related to growth and metabolism.

**Conclusion:** This study revealed large variability in CNVs and CNVRs across three Nellore lines differentially selected for YW and RFI. Gene annotation and gene ontology analyses of the exclusive CNVRs to each line revealed specific genes and biological processes involved in the expression of growth and feed efficiency traits. These findings contribute to the understanding of the genetic mechanisms underlying the phenotypic differences among the three Nellore selection lines.

## 1 Introduction

Nellore cattle (*Bos taurus indicus*) is the main beef cattle breed raised in Brazil, i.e., one of the largest beef producers and exporters in the world (United States Department of Agriculture, 2023). Nellore animals are well adapted to harsh climatic conditions and Brazilian herds have experienced major genetic progress for performance traits over the past decades (Fernandes Junior. et al., 2022). In addition to the national Nellore breeding programs, an experimental breeding program was initiated in 1980 in the Institute of Animal Science (IZ; Sertãozinho, SP, Brazil), with the establishment of three selection lines. At the beginning of the breeding program, the primary goal of the experiment was to assess the response to selection for heavier weights in a tropical beef cattle population (Mercadante et al., 2003). Briefly, the three selection lines were established by randomly dividing the founder animals into three groups: Nellore Control (NeC), Nellore Selection (NeS), and Nellore Traditional (NeT). NeC was maintained under stabilizing selection, in which animals with a yearling weight (YW) close to the average of the contemporary group were selected for breeding each year. NeS and NeT were selected for higher selection differentials for YW, and in 2008, residual feed intake (RFI) was also introduced as a selection criterion in the NeT line (Mercadante et al., 2003; Cardoso et al., 2018; Benfica et al., 2020).

After more than 40 years of selection, there are clear phenotypic and genetic differences among the lines subjected to stabilizing and directional selection. Cardoso et al. (2018) reported average yearling weight (YW) for males of 275 kg for the NeC line, 350 kg for the NeS line, and 360 kg for the NeT line, and Benfica et al. (2020) also reported average EBV for YW of 14.5 kg for NeC, 69.3 kg for NeS, and 72.2 kg for NeT, highlighting substantial phenotypic differences for YW between the three selection lines. Besides YW, substantial differences have been observed in other traits such as average body weight at different ages, body measurements, RFI, scrotal circumference, and carcass quality (Mercadante et al., 2003; Monteiro et al., 2013; Ceacero et al., 2016). Therefore, these three lines are a valuable resource for identifying genomic regions related to selection signatures, offering insights into the genes governing the phenotypic expression of these traits. Several studies have delved into the genetic mechanisms underlying phenotypic variations among these Nellore lines. For instance, genome-wide association studies (GWAS) have pinpointed key genes associated with growth and feed efficiency traits, while population genetic stratification has highlighted autosomal genomic regions exhibiting selection footprints (Ayres et al., 2010; Souza et al., 2011; Cardoso et al., 2018). Additionally, a new approach that could be further explored is the copy number variation (CNV), since artificial selection for desired traits has also been reported to impact the number of CNVs in animal genomes (Seol et al., 2019; Shi et al., 2023). For instance, a previous study has reported 3,161 CNVs and 561 CNV regions (CNVRs) in Nellore cattle, in which various CNVRs were significantly associated with dry matter intake and frequency of visits to the feed bunk (Benfica et al., 2024).

Copy Number Variations are structural variations within an individual’s genome, involving the loss or gain of DNA fragments, which can range from 1 kilobase pairs (kb) to several megabases (Mb) in size when compared to the reference genome of the species (Henrichsen et al., 2009). CNVs span extensive chromosomal regions and can change gene structure, regulatory modifications, gene dosage, and exposure of recessive alleles, leading to significant impact on gene expression (Zhang et al., 2009; Stafuzza et al., 2019) and phenotypic variability in complex traits (Zhang et al., 2009). The study of CNVs serves as a valuable source of information to elucidate some of the biological mechanisms contributing to the differences among the three experimental selection lines and in the phenotypic variations observed in economically important traits. Genetic selection for specific traits can lead to differential changes in allele frequencies across populations, and consequently, alterations in the genome of the animals (Bickhart et al., 2016; Buffalo and Coop, 2020; Das et al., 2021). CNV is a type of genome structural change that could drive phenotypic variation, evolution, and adaptation in populations under selection (Redon et al., 2006; Zhang et al., 2009; Lemos et al., 2018). Therefore, direct selection for weight gain may have shaped the landscape of CNVs in the genome of the cattle cattle lines with directional selection. Hence, the primary objectives of this study were to: 1) identify and characterize CNVs and CNVRs in Nellore cattle from three differentially-selected lines; and, 2) perform functional enrichment analyses of the identified CNVRs.

## 2 Materials and methods

### 2.1 Animals and experimental breeding program design

Data were collected from 928 animals, including 114 from the NeC line, 245 from the NeS line, and 569 from the NeT line. These animals were born between 2004 and 2019 and are part of the Nellore cattle herd from the Institute of Animal Science (IZ) in Sertãozinho, SP, Brazil. The animals are part of an experimental breeding program initiated in 1980 and separated into three selection lines: NeC, NeS, and NeT. These three lines are considered closed lines (Mercadante et al., 2003). Bulls were chosen from contemporary groups (defined by line and year) based on their YW adjusted to 378 days (W378) after a 168-day feedlot performance test. Replacement females, on the other hand, were selected based on their YW adjusted to 550 days (W550) while kept on pasture.

In the NeC line, males and females with a selection differential close to zero for YW were retained for breeding. Animals from the NeC line have maintained YW values that are close to the average observed at the outset of the breeding program in 1980. In contrast, for the selected NeS and NeT lines, both males and females with higher adjusted weights were selected over time. Starting in 2008, the bulls from the NeT line have been selected based on higher genomic estimated breeding values (GEBV) for YW and lower GEBV for RFI (more feed efficient animals) (Mercadante et al., 2003; Cardoso et al., 2018; Benfica et al., 2020). RFI was estimated as the residual of the linear regression equation of dry matter intake (DMI) on average daily gain (ADG) and mid-test metabolic weight (BW0.75) (Koch et al., 1963) in each test group.

**FIGURE 1:**
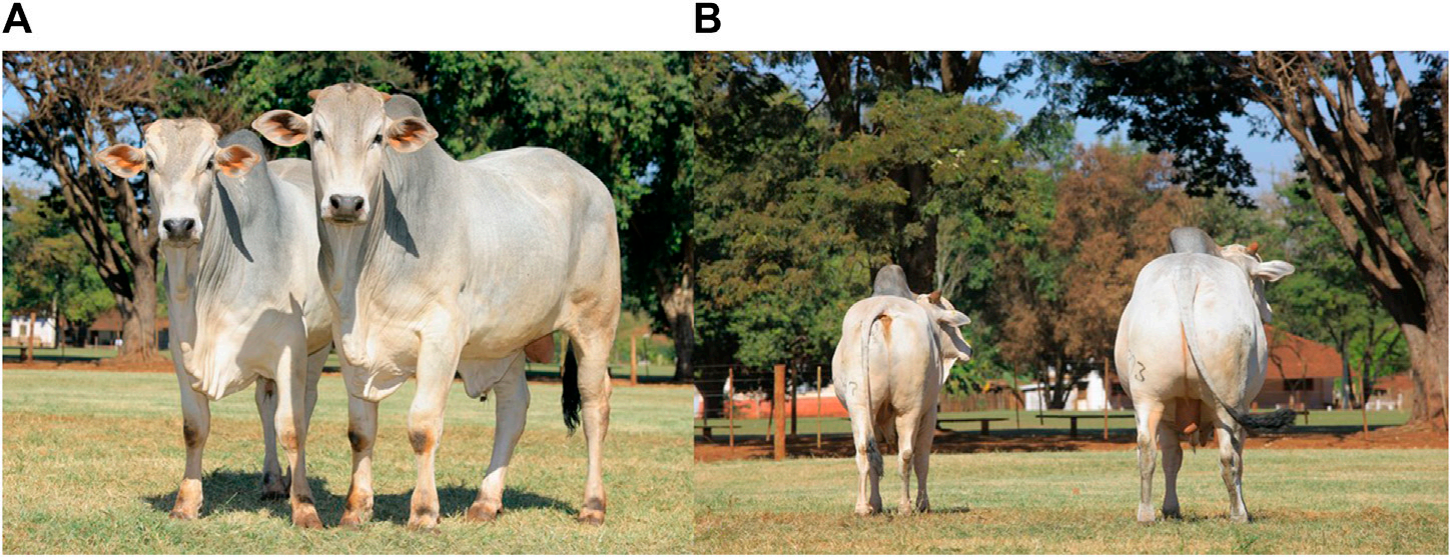
**(A,B)** Four-year-old sires from two differentially selected Nellore lines. NeS (right) and NeC (left) (Institute of Animal Science, 2020).

The sire selection strategy has been consistently applied to this day, involving the annual replacement of 50% of the three-year-old sires within each line. Furthermore, the annual culling rate for cows is approximately 20%. Figure 1 illustrates the differentiation in the phenotypic performance of the lines achieved through selection.

### 2.2 Genomic datasets

A total of 928 Nellore animals, including 625 males and 303 females, were genotyped with the Illumina BovineHD BeadChip (HD, Illumina Inc., San Diego, CA, United States; *n* = 770) or GeneSeek Genomic Profiler 50K (50K, GeneSeek Inc., Lincoln, NE, United States; *n* = 158) SNP panels. Approximately 75% of animals from the NeC line, 79% from the NeS line, and 86% from the NeT line were genotyped using the HD SNP panel (Supplementary Material S1). The HD and 50K SNP panels contained 777,962 and 54,791 SNPs, respectively, distributed throughout the genome. The mean distance between markers in the HD SNP panel was approximately equal to 3.43 ± 4.4 kilobases (Kb), while in the 50K panel, it was 49.2 ± 99.1 Kb. To ensure genomic data quality, non-autosomal SNPs, SNPs with an unknown genomic position, and SNPs with a GenCall score below 0.15 were removed during the quality control step. After the quality control process, 734,593 and 51,613 SNPs remained for subsequent analyses in the HD and 50K SNP panels, respectively.

### 2.3 Identification of copy number variation

The CNV identification was carried out separately for each SNP panel dataset using the PennCNV.1.0.5 software (Wang et al., 2007). This software integrates Log R Ratio (LRR) and B Allele Frequency (BAF) data on a per-sample basis into a hidden Markov model to determine the number of copies and genotypes of each CNV. LRR measures the total signal intensity, while BAF measures the proportion of the B allele in each sample. The population frequency of the B allele was calculated using the BAF value of each SNP in all samples. Furthermore, the LRR values were adjusted for the guanine-cytosine content at 500 kb upstream and downstream of each SNP based on a regression model (Diskin et al., 2008). This correction aims to reduce waviness that may result from the correlation between LRR and guanine-cytosine content in genomic regions, which could interfere with CNV detection.

Following CNV calling, a sample-based quality control process was implemented. This quality control step entailed the removal of CNVs with a BAF drift of less than 0.01, a standard deviation of LRR exceeding 0.30, a minimum length of 1,000 bp, a maximum length of 5,000,000 bp, and GC wave factor less than 0.05 (after genomic wave correction based on guanine-cytosine content). CNVs with less than three consecutive SNPs were also discarded. After this quality control, 883 animals and 14,914 CNVs (14,391 from the HD panel and 523 from the 50K panel) remained for further analyses. The CNVs identified were categorized and separated into the three distinct selection lines. This segregation led to the creation of distinct CNV datasets for each line, which were then utilized for conducting line-specific analyses. This approach enabled a thorough evaluation of CNVs within each selection line, providing valuable insights into the genetic diversity and potential functional significance of CNVs in these Nellore lines.

### 2.4 Identification of copy number variation regions

The CNVR were defined by grouping CNVs that had at least 1 bp overlap (Yan et al., 2015; Ma et al., 2017; Yang et al., 2017; Zhou et al., 2020; Zhou et al., 2022) using the *mergeBed* option of the BEDtools suite tool (Quinlan and Hall, 2010). This approach was applied in two contexts: across the entire population and within the specific selection lines being studied. CNVRs were classified as “loss” when an animal displayed a region with a loss of a chromosomal segment in comparison to the reference genome (deletions), “gain” for repeated chromosomal regions (duplications), and “mixed” when both loss and gain were identified within the same genomic region. Furthermore, CNVRs that were present in at least 10% of each line were identified. The CNVs and CNVRs were also identified separately for each selection line and compared across lines. An analysis of the overlapping CNVRs from each line was performed, and common and exclusive regions were identified.

### 2.5 Gene annotation and functional analyses

The CNVRs exclusive to each line were used for annotation purposes. The gene and QTL annotation in these regions were performed using the GALLO package (Fonseca et al., 2020), utilizing annotated data for *Bos taurus* retrieved from the Ensembl database (www.ensembl.org/Bos_taurus/Info/Index) and reference genome ARS-UCD1.2 (Rosen et al., 2020). Additionally, the Cattle QTL database (www.animalgenome.org/cgi-bin/QTLdb/BT/index) was used as a resource for obtaining previously-reported QTL information. The gprofiler2 package (Kolberg et al., 2020) was used for conducting Gene Ontology (GO) and KEGG pathway enrichment (*p* < 0.05) analyses to identify biological processes, molecular functions, cellular components, and biological pathways associated with the positional candidate genes identified.

## 3 Results

Table 1 presents descriptive statistics of all the animals from the three selection lines included in this study. The NeT line comprises the largest number of animals, followed by NeS and NeC. W378 ranged from 298 kg for NeC to 382 kg for NeS. In the case of W550, NeT had the highest average weight (363 ± 28 kg). Furthermore, the NeC line had the lowest average RFI (−0.112 ± 0.53 kg/day), followed by NeS (−0.032 ± 0.61 kg/day) and NeT (0.032 ± 0.60 kg/day).

**TABLE 1:** Descriptive statistics for Nellore Control (NeC), Nellore Selection (NeS), and Nellore Traditional (NeT).

		Trait^a^		
Selection Line	Number of animals	W378 (kg)	W550 (kg)	RFI (kg dry matter/day)
NeC	114	298 ± 41	266 ± 22	−0.112 ± 0.53
NeS	245	382 ± 46	336 ± 29	−0.032 ± 0.61
NeT	569	370 ± 46	363 ± 28	0.032 ± 0.60

^a^ W378, Yearling weight adjusted to 378 days; W550, Yearling weight adjusted to 550 days; RFI, residual feed intake.

### 3.1 Copy number variation and CNVR detection for the Nellore population

Initially, 20,259 CNVs were identified in 922 animals. After quality control, 14,914 CNVs located on autosomal chromosomes of 883 animals remained for further analyses, with an average of 16 CNVs per animal (range: 1–45). Among these identified CNVs, 3,680 were categorized as losses and 11,234 as gains. The length of the CNVs varied from 1,216 bp to 1,119,208 bp, with an average length of 75,632 ± 100,827 bp. Notably, CNVs were detected on all autosomal chromosomes and were non-uniformly distributed across the genome.

The 14,914 CNVs that remained after quality control were used to infer CNVRs by merging CNVs with at least a 1 bp overlap. This resulted in the identification of 1,884 CNVRs, with an average CNVR length of 40,887 ± 104,812 bp (range: 1,215 to 1,807,286 bp). Among these CNVRs, 400 of them were associated with genome losses, 1,412 with gains, and 72 with a mixed pattern, where the same chromosomal segment exhibited both deletion and duplication in the population. The number and proportion of chromosomes covered by CNVRs varied considerably (Table 2). BTA1 had the highest number of CNVRs (*n* = 181), covering 4.03% of the chromosome, while BTA12 had the highest coverage of a chromosome sequence (7.94%) with 107 CNVRs. In contrast, BTA25 had the lowest number of CNVR (*n* = 23) and BTA24 had the lowest coverage of a chromosome sequence at 0.87%. In total, the CNVRs identified in this study covered 77,031,673 bp of the autosomal genome sequence, which corresponds to approximately 3.09% of the cattle genome size.

**TABLE 2:** Chromosome distribution of all 1,884 copy number variation regions (CNVRs) detected in the Nellore cattle genome.

Chr^a^	Chr length (bp)	CNVR number	CNVR length (bp)	%^b^
BTA1	158,534,110	181	4,812,669	3.03
BTA2	136,231,102	88	3,278,939	2.41
BTA3	121,005,158	69	2,877,284	2.38
BTA4	120,000,601	94	3,077,289	2.56
BTA5	120,089,316	78	3,499,701	2.91
BTA6	117,806,340	108	5,347,225	4.54
BTA7	110,682,743	98	4,258,382	3.85
BTA8	113,319,770	82	2,149,785	1.89
BTA9	105,454,467	83	2,801,410	2.66
BTA10	103,308,737	61	4,176,247	4.04
BTA11	106,982,474	53	3,057,729	2.86
BTA12	87,216,183	107	6,927,991	7.94
BTA13	83,472,345	43	1,438,060	1.72
BTA14	82,403,003	82	3,206,441	3.89
BTA15	85,007,780	82	4,634,544	5.45
BTA16	81,013,979	66	2,632,004	3.25
BTA17	73,167,244	58	2,707,483	3.70
BTA18	65,820,629	43	996,000	1.51
BTA19	63,449,741	46	1,420,441	2.24
BTA20	71,974,595	49	2,085,746	2.89
BTA21	69,862,954	55	1,898,712	2.72
BTA22	60,773,035	30	700,159	1.15
BTA23	52,498,615	27	1,104,129	2.10
BTA24	62,317,253	32	541,048	0.87
BTA25	42,350,435	23	1,986,333	4.69
BTA26	51,992,305	39	1,278,351	2.46
BTA27	45,612,108	34	740,213	1.62
BTA28	45,940,150	29	1,102,340	2.39
BTA29	51,098,607	44	2,295,018	4.49
Total	2,489,385,779	1,884	77,031,673	3.09

^a^ Chromosome.

^b^ Percentage of the chromosome covered by CNVRs.

A noteworthy CNVR was identified in 847 animals, encompassing approximately 90% of the studied population (928 animals). This particular mixed type CNVR is located on BTA7, spanning a length of 1,133,904 bp. The gene content of this CNVR was thoroughly investigated, revealing an overlap with a total of 62 annotated genes (Supplementary Material S2).

The number and length of CNVs and CNVRs identified per SNP panel (50K and HD) were compared (Supplementary Material S1). The number of CNVs (50K: 523; HD: 14,391) and CNVRs (50K: 115; HD: 1,796) for the 50K SNP panel was higher compared to the HD SNP panel. Conversely, the average length of CNVs (50K: 114.4 ± 103 kb; HD: 74.2 ± 100 kb) and CNVRs (50K: 121.3 ± 129 kb; HD: 36.2 ± 96 kb) was smaller for the HD panel.

### 3.2 Copy number variation and CNVR detection by selection line

The 14,914 identified CNVs were categorized based on their respective selection lines, resulting in 1,510 CNVs in NeC animals, 3,899 CNVs in NeS, and 9,448 CNVs in NeT. The average CNV length were similar across the three selection lines, ranging from 71,886 ± 97,489 bp in NeC to 78,724 ± 102,183 bp in NeS. In all three lines, the number of loss type CNVs exceed that of gain CNVs, and the average (SD) number of CNVs per animal were 13.9 ± 7, 16.3 ± 8, and 17.6 ± 7 for NeC, NeS, and NeT, respectively. Detailed information about the CNVs per selection line after the quality control can be found in Table 3.

**TABLE 3:** Descriptive statistics of copy number variation (CNV) per Nellore selection line.

CNV type	Number	Min (bp)	Mean (bp)	Max (bp)	Min SNPs	Mean SNPs	Max SNPSs	Mean CNVs per animal
NeC (*n* = 108 animals)								
Loss	321	1,359	53,322	1,029,591	3	7.74	125	13.9
Gain	1,189	1,871	76,897	794,561	3	17.1	160	
Total	1,510	1,359	71,886	1,029,591	3	15.1	160	
NeS (*n* = 239 animals)								
Loss	1,023	1,359	85,879	766,200	3	9.48	64	16.3
Gain	2,876	1,454	76,179	883,985	3	17	175	
Total	3,899	1,359	78,724	883,985	3	15	175	
NeT (*n* = 536 animals)								
Loss	2,313	1,274	73,236	739,884	3	8.78	90	17.6
Gain	7,135	1,216	75,292	1,119,208	3	16.9	238	
Total	9,448	1,216	74,789	1,119,208	3	14.9	238	

NeC, Nellore control; NeS, Nellore selection; NeT, Nellore traditional.

**FIGURE 2:**
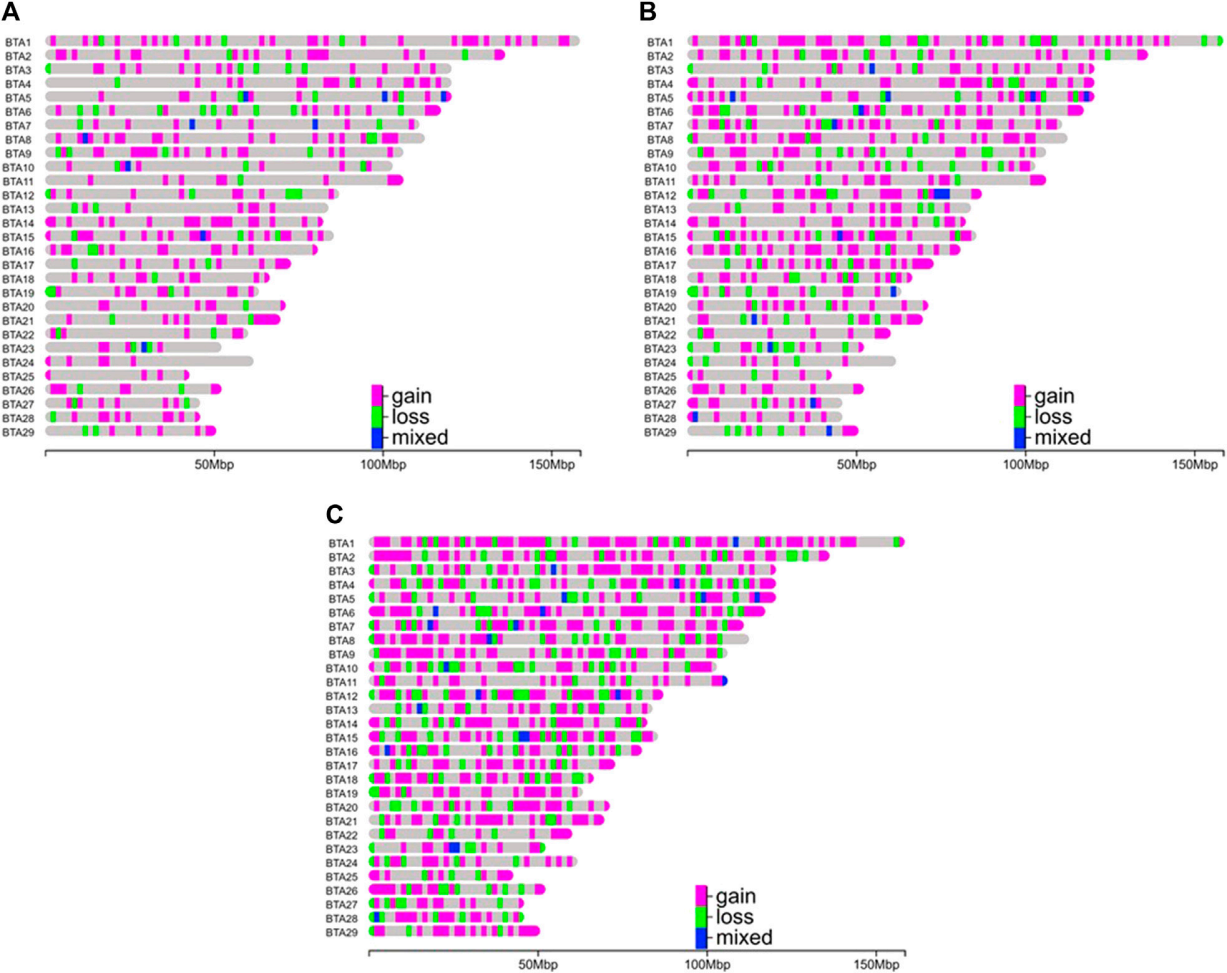
Distribution of copy number variation regions (deletions or losses, duplications or gains, and mixed type) by chromosome and selection line. **(A)** Nellore Control (NeC); **(B)** Nellore Selection (NeS); **(C)** Nellore Traditional (NeT).

The CNVRs were non-uniformly distributed across the chromosomes of the three Nellore lines (Figure 2). NeT had the highest number of CNVRs (*n* = 1,493), followed by NeS (*n* = 823) and NeC (*n* = 482). Among the three lines, BTA1 had the largest number of CNVRs, with 34 CNVRs identified in NeC, 81 in NeS, and 130 in NeT. On the other hand, BTA24 had the lowest CNVR count in both the NeC and NeS lines, with seven CNVRs in each line. NeT’s lowest CNVR count was observed on BTA25, with a total of 18 CNVRs. The CNVR coverage in the genomes of NeC, NeS, and NeT summed up to 23,449,890 bp, 40,175,556 bp, and 63,212,273 bp, respectively. This represents 0.94%, 1.61%, and 2.54% of the bovine autosomal genome for NeC, NeS, and NeT, respectively.

### 3.3 Common and exclusive CNVRs in the Nellore lines and gene annotation

**FIGURE 3:**
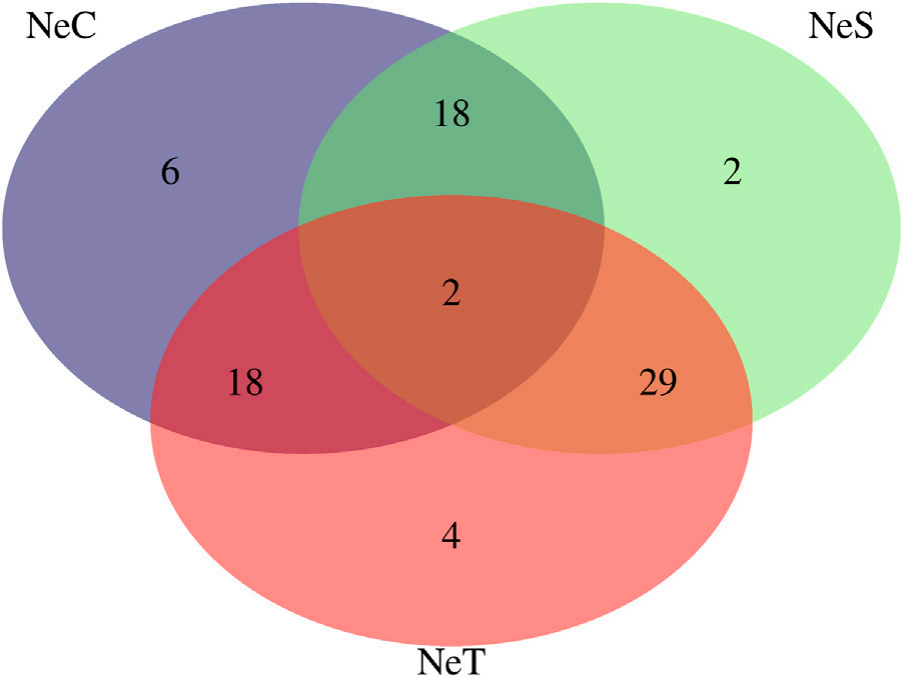
Venn Diagram for the copy number variation regions (CNVR) present in at least 10% of animals from Nellore Control (NeC), Nellore Selection (NeS), and Nellore Traditional (NeT) selection lines.

Twenty-five CNVRs, consisting of 6 losses, 4 gains, and 15 mixed type CNVRs, were identified in at least 10% of the NeC animals. In the NeS line, 32 CNVRs were observed, including 3 losses, 17 gains, and 12 mixed CNVRs. In the NeT line, 33 CNVRs were identified, with 4 losses, 18 gains, and 11 mixed CNVRs. The average length of these CNVRs was 283,307 ± 283,739 bp for NeC, 355,917 ± 290,815 bp for NeS, and 381,594 ± 354,594 bp for NeT. Interestingly, two CNVRs were commonly identified across all three selected lines. Additionally, there were 18 regions shared between NeC and NeS, 18 regions shared between NeC and NeT, and 29 regions shared between NeS and NeT, as illustrated in Figure 3. The two regions that were identified as common to all three lines overlapped with 11 annotated genes, as shown in Table 4.

**TABLE 4:** Description of the copy number variation regions (CNVR) commonly identified among the three selection lines.

CNVR	BTA^a^	Start	End	Type	Gene ensembl ID	Gene symbol
CNVR1	5	59,421,039	59,650,955	Mixed	*ENSBTAG00000052799*	
					*ENSBTAG00000054478*	*OR9K2I*
					*ENSBTAG00000038567*	*OR9K2H*
					*ENSBTAG00000045722*	*OR9K2K*
					*ENSBTAG00000050988*	*OR9K2C*
					*ENSBTAG00000054855*	
CNVR2	6	113,462,586	113,603,960	Gain	*ENSBTAG00000005493*	*TBC1D14*
					*ENSBTAG00000047810*	*CCDC96*
					*ENSBTAG00000010185*	*TADA2B*
					*ENSBTAG00000010181*	*GRPEL1 bta-mir-2453*
					*ENSBTAG00000044374*	

^a^ *Bos taurus* autosomal chromosomes.

Regarding the exclusive regions, there were 6 CNVR identified for NeC, 2 regions for NeS, and 4 regions for the NeT line. Out of the 6 exclusive CNVRs in the NeC line, there were 3 loss type CNVR and 3 gain type CNVR, distributed across 6 chromosomes, with an average length of 91,745 ± 119,203 bp. Out of the 6 CNVRs, 3 of them overlapped with 16 annotated genes (Table 5).

**TABLE 5:** Description of the copy number variation regions (CNVR) identified exclusively in the Nellore Control line.

CNVR	BTA^a^	Start	End	Type	Gene ensembl ID	Gene symbol
CNVR3	3	25,683	82,937	Loss	*ENSBTAG00000047047*	*OR5W25P*
CNVR4	4	113,657,341	113,819,352	Gain	*ENSBTAG00000011229*	
					*ENSBTAG00000048379*	*TMUB1*
					*ENSBTAG00000008468*	*AGAP3*
					*ENSBTAG00000016752*	*ASB10*
					*ENSBTAG00000011120*	*GBX1*
					*ENSBTAG00000018838*	*IQCA1L*
					*ENSBTAG00000024204*	*H2BK1*
					*ENSBTAG00000000607*	*ABCF2*
					*ENSBTAG00000014371*	*CHPF2 bta-mir-671*
					*ENSBTAG00000036355*	*SMARCD3*
					*ENSBTAG00000014372*	
CNVR5	8	38,362,326	38,537,300	Gain	*ENSBTAG00000020815*	*UHRF2*
					*ENSBTAG00000011161*	*TPD52L3*
					*ENSBTAG00000011160*	*IL33*
					*ENSBTAG00000018347*	
CNVR6	9	4,374,671	4,386,831	Loss		
CNVR7	13	7,963,451	8,013,473	Loss		
CNVR8	18	18,822,010	18,843,543	Gain		

^a^ *Bos taurus* autosomal chromosomes.

In the case of the NeS line, there were two exclusive CNVRs, and both of these regions were classified as mixed type, indicating both deletions and duplications. These CNVRs were found on BTA12, with an average length of approximately 812,093 ± 147,962 bp each. Notably, both CNVRs were identified within genomic regions in the reference genome assembly and overlapped with 8 genes, as shown in Table 6.

**TABLE 6:** Description of the copy number variation regions identified exclusively in the Nellore Selection line.

CNVR	BTA^a^	Start	End	Type	Gene ensembl ID	Gene symbol
CNVR9	12	71,274,536	72,191,254	Mixed	*ENSBTAG00000026070*	
					*ENSBTAG00000052990*	
					*ENSBTAG00000047181*	
CNVR10	12	75,238,779	75,946,247	Mixed	*ENSBTAG00000000146*	*FARP1*
					*ENSBTAG00000045262*	*U6*
					*ENSBTAG00000032234*	*STK24*
					*ENSBTAG00000004440*	*SLC15A1*
					*ENSBTAG00000010395*	*DOCK9*

^a^ *Bos taurus* autosomal chromosomes.

In the NeT line, there were two exclusive loss regions and two exclusive gain regions, distributed across four chromosomes (BTA1, BTA6, BTA17, BTA21). The average length of these exclusive CNVRs was approximately 233,107 ± 279,300 bp. Among these regions, three overlapped with 21 genes, as presented in Table 7.

**TABLE 7:** Description of the copy number variation regions (CNVR) identified exclusively in the Nellore Traditional line.

CNVR	BTA^a^	Start	End	Type	Gene ensembl ID	Gene symbol
CNVR11	1	15,986,400	15,996,851	Loss		
CNVR12	6	114,086,816	114,716,539	Gain	*ENSBTAG00000027434*	
					*ENSBTAG00000012464*	*SORCS2*
					*ENSBTAG00000015926*	*AFAP1*
					*ENSBTAG00000036422*	*ABLIM2 bta-mir-95*
					*ENSBTAG00000047876*	*SH3TC1*
					*ENSBTAG00000047613*	*HTRA3*
					*ENSBTAG00000055218*	*ACOX3*
					*ENSBTAG00000004893*	
CNVR13	17	71,806,249	72,029,884	Gain	*ENSBTAG00000017277*	*RAB36*
					*ENSBTAG00000036113*	*RSPH14*
					*ENSBTAG00000047664*	*GNAZ*
					*ENSBTAG00000023007*	*VPREB1*
					*ENSBTAG00000055118*	*VPREB1*
					*ENSBTAG00000052191*	*TOP3B*
					*ENSBTAG00000053372*	*PPM1F*
					*ENSBTAG00000006938*	*MAPK1*
					*ENSBTAG00000010312*	*U6*
					*ENSBTAG00000042240*	
CNVR14	21	20,062,038	20,130,658	Loss	*ENSBTAG00000001526*	
					*ENSBTAG00000053783*	
					*ENSBTAG00000050647*	

a *Bos taurus* autosomal chromosomes.

### 3.4 Gene ontology and QTL identification

The genes that overlapped with exclusive CNVRs from each selection line were included in the gene ontology (GO) analyses. While the functional analyses of genes conducted using the gprofiler2 package (Kolberg et al., 2020) did not yield significant results for the NeC and NeT cattle lines, a closer investigation of the functions of biological processes associated with these genes revealed their involvement in specific biological pathways. These genes were involved in pathways such as thermogenesis (NeC), fatty acid metabolism (NeC), and protein digestion and absorption (NeS). For the NeT line, functional enrichment was observed in the cellular component category, specifically for the term GO:0016020—Integral component of membrane. Genes within the exclusive regions of NeT also contribute to various biological processes, including positive regulation of growth (GO:0045927), positive regulation of gene expression (GO:0010628), and insulin-like growth factor receptor signaling pathway (GO:0048009). Furthermore, these genes play important roles in metabolic pathways related to growth hormone synthesis and secretion. Within the exclusive CNVRs of each selection line, the number of previously reported QTL overlapping with the genomic regions identified for NeC, NeS, and NeT were 12, 27, and 146, respectively. Among these, 8 QTL previously associated with production traits (e.g., ADG) overlap with the NeC regions, 2 QTL associated with production (e.g., ADG and maturity rate) for NeS, and 2 QTL associated with production traits (body weight gain and metabolic body weight) in NeT (Supplementary Material S3).

## 4 Discussion

The Nellore experimental breeding program from IZ has gained national recognition and contributed substantially to the field of beef cattle breeding and genetics. The differential selection among the three selection lines has enabled in-depth studies of weight-related traits and feed efficiency, providing essential insights into the genetic information of livestock (e.g., Ayres et al., 2010; Cardoso et al., 2014; Cardoso et al., 2018).

The NeC line had the lowest average for W378 and W550, which was expected since this line is characterized by stabilizing selection with an average YW close to the weight at the start of the breeding program. The NeS line exhibited the highest mean for W378, which aligns with this line’s selection focus on increased post-weaning weight, highlighting the success of the breeding program in attaining its specific breeding objective. Considering the substantial difference in the average of W378 and W550 between lines, the three lines provide a great opportunity to identify genomic regions altered by selection. The NeC animals can be used as a reference point to compare the lines and understand the genetic progress achieved over time and the mechanisms involved in the phenotypic expression of the selected traits. NeC exhibited the lowest phenotypic average for RFI (more efficient), followed by NeS and NeT. However, it is important to highlight that the standard deviations (SD) were high for these averages, and these values are representative of only a small subset of Nellore animals, thus not accurately reflecting the population mean of each line.

### 4.1 Copy number variation and CNVR detection in Nellore cattle

Numerous studies have previously investigated the distribution and characterization of CNVs and CNVRs within the cattle genome (e.g., Fadista et al., 2010; Liu et al., 2010; Hou et al., 2012; Peripolli et al., 2023), each yielding diverse findings and insights about the presence and the function of these variants in the cattle genome. For instance, Silva et al. (2016) identified 68,007 CNVs and 7,319 CNVRs in a population of 1,509 Nellore animals. Additionally, Upadhyay et al. (2017) reported 9,944 CNVs and 923 CNVRs in 149 European cattle, while Lemos et al. (2018) identified 195,873 CNVs and 9,805 CNVRs in 3,794 Nellore animals. In a study of Holstein cattle, Butty et al. (2021) found 23,256 CNVs and 1,645 CNVRs. There is a clear notable discrepancy in the number of CNVs and CNVRs between the previously reported study and our current findings. However, each study utilized different SNP panel densities, quality control thresholds, and sample sizes, which may have contributed to these differences (Fadista et al., 2010; Hou et al., 2012). Furthermore, the implementation of quality control measures, accounting for batch effects, addressing population stratification, managing experimental variations, and the robustness of statistical models can all impact the detection and accuracy of CNVs (Dellinger et al., 2010). Therefore, any comparisons between studies should be made cautiously, considering all these factors described above. The proportion of the genome covered by CNVRs (3.09%) falls within the range reported in the literature. Previous studies have reported values ranging from 0.68% to 13.0% in cattle populations (Fadista et al., 2010; Zhou et al., 2016; Lemos et al., 2018).

The distribution of CNVRs across chromosomes did not follow any clear pattern and BTA1 exhibited the highest number of CNVRs (*n* = 181), a trend also noted by Silva et al. (2016). Although no particular pattern or correlation was observed, this result may be associated with the fact that BTA1 is the largest chromosome in the cattle genome. Another interesting finding in the present study was the identification of a CNVR present in 90% of the individuals included in the study. This observation suggests the existence of a region that has remained conserved within this Nellore population over time, highlighting potential genetic stability or selection pressure within this genomic region. This might also reflect the fact that the reference genome used was based on a taurine (*Bos taurus taurus*) animal while Nellore is a different subspecies (*Bos taurus indicus*). This highlights the need to develop cattle pangenomes (e.g., Zhou et al., 2022).

This common region observed in 90% of the studied population is a gene-rich region containing 62 annotated genes. Several genes associated with male and female reproductive traits were identified, including *THEG* (Nayernia et al., 1999; Mannan et al., 2003), *FGF22* (Castilho et al., 2017; 2019), *KISS1R* (D’Occhio et al., 2020; Singh et al., 2020), and *ARID3A* (Yang et al., 2018). Furthermore, genes linked to the immune system such as *AZU1* (Xu et al., 2018; Verardo et al., 2021) and *ELANE* (Cassatella et al., 2019; Verardo et al., 2021) were also identified. The *CFD* gene was also previously associated with fat accumulation (Wang et al., 2023) and overlapped with the region cited above.

It is important to note that the present study utilized two genotyping panels of different densities for the CNV analyses, including one with 777,962 SNPs and one with 54,791 SNPs. Although 83% of the animals used in this study were genotyped with the HD SNP panel, the use of the 50K SNP panel may be considered as a limitation of the study. Genotyping panels with higher density contain a greater number of genomic markers distributed throughout the genome, and generally enable more accurate detection of CNVs with higher genomic location resolution (Wang et al., 2007). This may explain why the number of CNVs and CNVRs found was higher for the HD SNP panel while their length was shorter as compared to the CNVs and CNVRs identified based on the 50K data. The use of a 50K SNP panel may impact CNV detection (e.g., longer CNVs may be incorrectly identified) and limit the ability to identify CNVs in genomic regions containing less SNPs after the quality control. Additionally, the number of animals genotyped with the HD SNP panel in this study is ∼5 times larger than the number of animals genotyped with the 50K SNP panel, which may also have contributed to the higher number of CNVs and CNVRs detected based on the HD SNP panel. In this study, no animals were genotyped with the same SNP panel to enable comparison of the results on an animal basis. Although out of the scope of this current study, future studies using genotyping platforms of different densities as well as molecular approaches for validating the identified CNVs are warranted. This will enable the evaluation of the impact of the SNP density on CNV detection.

### 4.2 Copy number variation and CNVR detection by line

While previous studies have identified CNVs within and between cattle populations, our study is one of the first endeavors to investigate the population-genetic properties in three closed Nellore lines that were differentially selected for high post-weaning weight and RFI. Substantial differences in CNV counts were identified among the three lines studied. NeS and NeT exhibited a relatively high number of CNVs and CNVs per individual compared to NeC, along with a high chromosome coverage by CNVRs. The results in this study are based on a population of 928 animals with an uneven distribution among the lines. However, for the purpose of comparison and confirmation of the results, CNVs and CNVRs were also identified considering a reduced number of animals with an equal number of samples per line (*n* = 114). Remarkably, the results remained consistent with the same pattern (results not shown), where animals from the NeS and NeT lines exhibited a higher number of CNVs and CNVRs.

The results obtained align with previous expectations and are supported by the findings from Upadhyay et al. (2017), who reported that the population size, gene flow, and the selection process in a population can contribute to differential CNV abundance among populations. Selection for a specific trait can indeed lead to changes in allele frequencies within the population, resulting in alterations within the cattle genome and giving rise to significant phenotypic and genetic variability (Bickhart et al., 2016). Furthermore, the present findings are consistent with the results of Strillacci et al. (2018), who reported CNVs and CNVRs within the genome of Valdostana Red Pied cattle, an Italian dual-purpose cattle population that did not undergo strong artificial selection for production traits. Following the CNV identification, the authors conducted a comparative analysis of the CNVs detected in their study with those available from published research in the Italian Brown Swiss and Mexican Holstein populations (Strillacci et al., 2018). Their findings revealed the presence of unique and highly differentiated CNVs, leading to the conclusion that directional selection occurring within a population exerts a significant impact on the genome in terms of CNVs.

Despite differences in the numbers of CNVs identified, all three selection lines exhibited a higher frequency of duplications than deletions. This observation aligns with findings from previous studies, such as Laseca et al. (2022) in horses, Ladeira et al. (2022) in sheep, and Liu et al. (2010) in cattle. While there is no clear pattern of duplication and deletion distribution across the genome, duplications are more likely to occur in CNVs with greater lengths (Locke et al., 2006). Furthermore, according to Amos et al. (2003) and Conrad et al. (2006), deletion events may go unnoticed using SNP genotyping methods.

### 4.3 Gene annotation, gene ontology, and QTL identification

The deletion or duplication of genomic regions can have various consequences. The deletion of a genomic region that contains important genes can lead to the loss of gene function, potentially being associated with diseases, genetic disorders, and reduced fitness (Stenson et al., 2017). Moreover, the duplication of gene-rich regions may also be associated with adaptation (Sharma et al., 2018; Meredith et al., 2024). On the other hand, the duplication of gene-rich regions is typically linked to genetic diversity. Gene duplication is believed to play an important role in evolution and adaptation and may be involved in the development of new gene functions (Zhang, 2003; Magadum et al., 2013; Lallemand et al., 2020). Thus, we identified genes present in exclusive regions for each selection line, which may help elucidate differences between lines and the expression of traits in a selection process.

Gene ontology analysis is also an essential tool for elucidating the functional landscape of genetic elements, as it helps to comprehend and interpret the functions of genes. In the current study, no enrichment of biological processes was observed for the genes identified. This suggests that collectively, they do not participate in any similar biological process, potentially indicating a diverse array of gene functions. However, even though enriched processes were not identified, the genes individually participate in crucial biological processes and pathways. These findings suggest that while there may not be overall enriched processes, the individual genes within these regions may collectively contribute to the regulation of vital biological processes associated with growth and gene expression.

In the NeC line, the CNVR4 is a gain region that harbors 11 genes and 12 QTL. Within this genomic region, the gene *SMARCD3* stands out as it overlaps with 8 previously reported QTL that are related to ADG. The *SMARCD3* gene plays a crucial role as a subunit of the SWI/SNF family of proteins, which are known for their helicase and ATPase activities and their capacity to modulate the transcription of specific genes by modifying the chromatin structure surrounding those genes. ATPase is an enzyme that catalyzes the hydrolysis of ATP (adenosine triphosphate), releasing energy that is utilized in a variety of cellular processes, including ion transport, macromolecule synthesis, and muscular contraction (Rappas et al., 2004; Hargreaves and Spriet, 2020). Therefore, the activity of ATPase can influence the energy metabolism and, consequently, ADG and body weight gain of animals. The fact that the *SMARCD3* overlaps with 8 QTL related to ADG is a significant finding, suggesting a potential functional relationship between this gene and ADG and YW. This indicates that the CNVR4 might be directly involved in the expression of the trait, potentially explaining some of the phenotypic differences observed between the NeC line and the NeS and NeT lines. Additionally, the *SMARCD3* gene has been linked to biological processes related to muscle cell differentiation and thermogenesis pathways. Muscle cell differentiation is essential for the development of animal muscle tissue (Purslow, 2022) and the efficiency in the muscle cell differentiation process can affect the rate and magnitude of weight gain. Thermogenesis is also an important process that can impact animal weight as it is essential for maintaining body temperature and basal metabolism (Hhmms-Hagen, 1989; Cannon and Nedergaard, 2011). Considering that thermogenesis is linked to energy expenditure, it is plausible that it may also influence the ADG of animals, and consequently body weight at specific time points (e.g., YW). Another important NeC region is the CNVR5 on BTA8, which contains the *UHF2* gene. This gene encodes a nuclear protein involved in cell-cycle regulation (Lu and Hallstrom, 2013). The *UHF2* gene has been reported to be involved in the regulation of many biological processes, including metabolic pathways, growth, and reproduction (Magoro et al., 2022).

In the NeS line, the CNVR10 located on BTA12 overlaps with the *SLC15A1* gene. This gene encodes an intestinal hydrogen peptide cotransporter and belongs to the solute carrier family 15. *SLC15A1* plays a crucial role in the uptake and digestion of dietary proteins (Liang et al., 1995). Additionally, *SLC15A1* has been associated with small intestine weight and embryo development in chickens (Zeng et al., 2011; Li et al., 2013) as well as with protein digestion and absorption pathways. Efficient digestion and absorption of proteins are essential to ensure that cattle receive the necessary nutrients and can affect the growth and weight gain of animals (Pierzynowski et al., 2006). Furthermore, a QTL related to ADG also overlapped with CNVR10. This evidence suggest the potential significance in regulating critical processes related to nutrient absorption, intestinal development, and overall growth in cattle. Another noteworthy point is that despite only one QTL related to ADG being identified in the NeS line, a total of 20 QTL related to milk production traits were identified in CNVR9 and CNVR10, and associations between milk production and YW have been previously reported (e.g., Lee and Pollak, 2002; Gershoni et al., 2021).

In the NeT line, several exclusive regions overlapping with important genes were identified. One of these regions, CNVR13, stood out as a gain type CNVR located on BTA17. This region encompasses 10 genes, with particular emphasis on *MAPK1*. *MAPK1* encodes a member of the MAP kinase family. MAP kinases, also known as extracellular signal-regulated kinases, serve as a central hub for integrating multiple biochemical signals and play integral roles in a wide array of cellular processes, including proliferation, differentiation, transcription regulation, and development (Jiang et al., 2011; Liu et al., 2016). Moreover, previous studies have reported that the *MAPK1* gene is linked to cell growth in phosphorylation and protein modification process, which are needed for the muscle growth mechanism (Shin et al., 2014). Furthermore, the *MAPK1* gene is associated with biological processes related to Insulin-like growth factor receptor signaling pathway and growth hormone synthesis pathways. These processes play a pivotal role in the growth and development of cattle. Growth hormone synthesis and Insulin-like growth factor are crucial for regulating energy metabolism, adipose tissue deposition, and muscle growth, ensuring adequate animal weight gains (Dichtel et al., 2022; Zhang et al., 2022). The *MAPK1* gene is also associated with the biological process term GO:0010628, defined as positive regulation of gene expression. Another gene identified in this region is *PPM1F.* Although no significant results were found in the GO analyses, *PPM1F* gene is related to biological terms associated with growth factors (GO:0045927, defined as positive regulation of growth).

Another important region identified for the NeT line is the CNVR12, located on BTA6, which overlaps with eight genes and QTL related to body weight gain, metabolic body weight, and carcass weight. The *ACOX3* gene within this region has been associated with metabolic pathways related to fatty acid degradation and fatty acid metabolism. Fatty acid metabolism is directly linked to energy regulation, fat storage, and overall lipid metabolism. Efficient fatty acid degradation can contribute to energy release and the maintenance of adequate energy balance (Miyamoto et al., 2016), which is essential for controlling body weight and vital biological functions.

Considering the significant phenotypic differences observed in YW among the three selections lines, it was expected to find differences in the identification of CNVs and CNVRs between the lines. The discovery of unique regions containing distinct genes, biological processes, pathways, and QTL related to the traits is an important finding. This suggests that the presence of these exclusive CNVRs may control the expression of phenotypes related to YW and feed efficiency and contribute to phenotypic response to selection. However, the studied populations were selected for quantitative traits, which are influenced by many genes (and genomic regions). Therefore, there are likely many other genes and genomic structural variations not identified in this study affecting the phenotypic variability on the traits under selection.

## 5 Conclusion

We described a variability of CNVs and CNVRs within three Nellore lines differentially selected for YW and RFI. Through the gene annotation and gene ontology analyses of the exclusive CNVRs identified in each line, specific genes and biological processes involved in the expression of growth and feed efficiency traits were found. These results not only show the structural differences present in the genomes of animals from the three studied selection lines but also indicate that these variations may account for a portion of the observed differences among them. These findings provide valuable insights for future research and breeding strategies to enhance these important traits in Nellore cattle populations.

## Data Availability

The data analyzed in this study is subject to the following licenses/restrictions: The data supporting this study’s findings belongs to an experimental animal breeding program and can be made available by contacting the corresponding author upon reasonable request and with permission of the breeding program. Requests to access these datasets should be directed to MM, mezmercadante@gmail.com.
