# Supplementary material for: Detection and characterization of copy number variation in three differentially-selected Nellore cattle populations
Source: Front Genet. 2024 Apr 17;15:1377130. doi: 10.3389/fgene.2024.1377130 (PMC11061390; doi:10.3389/fgene.2024.1377130)
Supplement: Supplementary file 1 [file Table1.DOCX]

Supplementary Material 2

**Table S2.** List of the Copy Number Variation regions (CNVR) identified in more than 90% of the Nellore cattle individuals included in the analyses and their overlapping genes

| CNVR | BTA^a^ | Start | End | Type | Gene Ensembl ID | Gene Symbol |
| --- | --- | --- | --- | --- | --- | --- |
| CNVR | 7 | 42,718,008 | 43,851,912 | Mixed | *ENSBTAG00000019814*  *ENSBTAG00000046200*  *ENSBTAG00000046953*  *ENSBTAG00000009273*  *ENSBTAG00000045589*  *ENSBTAG00000049046*  *ENSBTAG00000052753*  *ENSBTAG00000052768*  *ENSBTAG00000048841*  *ENSBTAG00000000717*  *ENSBTAG00000019767*  *ENSBTAG00000048393*  *ENSBTAG00000047560*  *ENSBTAG00000016072*  *ENSBTAG00000039372*  *ENSBTAG00000050765*  *ENSBTAG00000039355*  *ENSBTAG00000010772*  *ENSBTAG00000002098*  *ENSBTAG00000002100*  *ENSBTAG00000016648*  *ENSBTAG00000015050*  *ENSBTAG00000015051*  *ENSBTAG00000027357*  *ENSBTAG00000014349*  *ENSBTAG00000003018*  *ENSBTAG00000021616*  *ENSBTAG00000019771*  *ENSBTAG00000020269*  *ENSBTAG00000045828*  *ENSBTAG00000051609*  *ENSBTAG00000045829*  *ENSBTAG00000049418*  *ENSBTAG00000046105*  *ENSBTAG00000046188*  *ENSBTAG00000048122*  *ENSBTAG00000047217*  *ENSBTAG00000053281*  *ENSBTAG00000046406*  *ENSBTAG00000037418*  *ENSBTAG00000008607*  *ENSBTAG00000002434*  *ENSBTAG00000025597*  *ENSBTAG00000011351*  *ENSBTAG00000020764*  *ENSBTAG00000020766*  *ENSBTAG00000020772*  *ENSBTAG00000020776*  *ENSBTAG00000053003*  *ENSBTAG00000020780*  *ENSBTAG00000011639*  *ENSBTAG00000025233*  *ENSBTAG00000000550*  *ENSBTAG00000012172*  *ENSBTAG00000007480*  *ENSBTAG00000046542*  *ENSBTAG00000038221*  *ENSBTAG00000030839*  *ENSBTAG00000019419*  *ENSBTAG00000004112*  *ENSBTAG00000019714*  *ENSBTAG00000019718* | *PGBD2*  *OR2AV1*  *OR2AV14*  *OR2AV2*  *OR2AV11*  *OR2AZ3*  *OR2AZ3B*  *OR2AZ1*  *PLPP2*  *MIER2*  *THEG*  *C2CD4C*  *SHC2*  *ODF3L2*  *MADCAM1*  *TPGS1*  *CDC34*  *GZMM*  *BSG*  *HCN2*  *POLRMT*  *FGF22*  *RNF126*  *FSTL3*  *PRSS57*  *PALM*  *MISP*  *PTBP1*  *PLPPR3*  *AZU1*  *U6*  *PRTN3*  *ELANE*  *CFD*  *MED16*  *U6*  *R3HDM4*  *KISS1R*  *ARID3A*  *WDR18*  *GRIN3B*  *TMEM259*  *CNN2*  *ABCA7*  *ARHGAP45*  *POLR2E*  *GPX4*  *SBNO2*  *STK11*  *CBARP*  *ATP5F1D*  *MIDN*  *CIRBP*  *FAM174C*  *EFNA2*  *PWWP3A*  *NDUFS7*  *GAMT*  *DAZAP1*  *RPS15* |

^a^Chromosome
