# Supplementary material for: Detection and characterization of copy number variation in three differentially-selected Nellore cattle populations
Source: Front Genet. 2024 Apr 17;15:1377130. doi: 10.3389/fgene.2024.1377130 (PMC11061390; doi:10.3389/fgene.2024.1377130)
Supplement: Supplementary file 2 [file Table2.DOCX]

Supplementary Material 3

**Table S3:** Quantitative trait loci (QTLs) identified in the unique regions of each selection line

| Chr^a^ | Start | End | N. QTL^b^ | QTL type | Trait ID (n)^c^ |
| --- | --- | --- | --- | --- | --- |
| NeC | | | | | |
| 4 | 113,657,341 | 113,819,352 | 12 | Production  Reproduction  Meat and carcass  Meat and carcass  Health | Average daily gain (8)  First service conception (1)  Tenderness score (1)  Connective tissue amount (1)  Somatic cell count (1) |
| NeS | | | | | |
| 12 | 71,274,536 | 72,191,254 | 9 | Health  Milk  Milk  Milk  Milk  Exterior  Milk  Milk  Milk | Paratuberculosis susceptibility (1)  Milk C16 index (1)  Milk capric acid content (1)  Milk lauric acid content (1)  Milk caprylic acid content (1)  Body condition score (1)  Milk fat percentage (1)  Milk protein percentage (1)  Somatic cell score (1) |
| 12 | 75,238,779 | 75,946,247 | 18 | Reproduction  Production  Health  Milk  Production  Meat and carcass  Milk  Milk  Milk | Pregnancy rate (1)  Average daily gain (1)  Dopamine level (1)  Milk fat percentage (1)  Maturity rate (1)  Longissimus muscle area (1)  Milk glycosylated kappa-casein percentage (10)  Milk myristic acid content (1)  Milk caprylic acid content (1) |
| NeT | | | | | |
| 6 | 114,086,816 | 114,716,539 | 8 | Milk  Reproduction  Milk  Production  Production | Milk iron content (3)  Inseminations per conception (1)  Milk kappa-casein percentage (2)  Metabolic body weight (1)  Body weight gain (1) |
| 17 | 71,806,249 | 72,029,884 | 137 | Reproduction  Meat and carcass  Exterior | Non-return rate (135)  Longissimus muscle area (1)  Udder cleft (1) |
| 21 | 20,062,038 | 20,130,658 | 1 | Meat and carcass | Carcass weight (1) |

^a^Chromosome

^b^Number of Quantitative Trait Loci (QTL)

^c^Number of trait type
