# Supplementary material for: Detection and characterization of copy number variation in three differentially-selected Nellore cattle populations
Source: Front Genet. 2024 Apr 17;15:1377130. doi: 10.3389/fgene.2024.1377130 (PMC11061390; doi:10.3389/fgene.2024.1377130)
Supplement: Supplementary file 3 [file Table3.DOCX]

Supplementary Material 1

**Table S1:** Number of genotyped animals, copy number variation, and copy number variation regions per SNP panel

|  | Number of animals | | | |  | CNV | |  | CNVR | | |
| --- | --- | --- | --- | --- | --- | --- | --- | --- | --- | --- | --- |
| Panel | Total | NeC | NeS | NeT |  | N^c^ | Length(kb)^d^ |  | N^c^ | Length(kb)^d^ | L:G^e^ |
| 50K | 158 | 25 | 51 | 82 |  | 523 | 114.4±103 |  | 115 | 121.3±129 | 0.24 |
| HD | 770 | 89 | 194 | 487 |  | 14,391 | 74.2± 100 |  | 1,796 | 36.2±96 | 0.29 |

^a^50K: GeneSeek Genomic Profiler 50K; HD: Illumina BovineHD BeadChip

^b^NeC: Nellore Control; NeS: Nellore Selection; NeT: Nellore Traditional

^c^N: Number of CNV/CNVR

^d^kb: kilo base pairs

^e^L:G: Proportion of deletion:duplication
